# A prognostic signature of cuproptosis and TCA-related genes for hepatocellular carcinoma

**DOI:** 10.3389/fonc.2022.1040736

**Published:** 2022-10-17

**Authors:** Qi Zhang, Longping Ma, Hongyuan Zhou, Yanzhao Zhou, Shuaijing Liu, Qiang Li

**Affiliations:** Department of Hepatobiliary Cancer, Liver Cancer Research Center, Tianjin Medical University Cancer Institute and Hospital, National Clinical Research Center for Cancer, Key Laboratory of Cancer Prevention and Therapy, Tianjin’s Clinical Research Center for Cancer, Tianjin, China

**Keywords:** cuproptosis, elesclomol, overall survival, hepatocellular carcinoma, tricarboxylic acid cycle (TCA cycle)

## Abstract

**Background:**

Hepatocellular carcinoma (HCC) is the most common malignant tumor of the liver. Cuproptosis is a newly defined form of cell death. Copper ion induces cell death by binding to the tricarboxylic acid cycle (TCA). The effect of cuproptosis-related and TCA-related genes on the clinical prognosis of HCC is still unclear. In this study, we explores the genetic changes of cuproptosis-related genes that affect the TCA process and their potential therapeutic value in HCC patients.

**Methods:**

The cuproptosis and TCA-related genes were obtained from cuproptosis-related articles and the molecular signatures database. The prognosis signatures of eight related genes were constructed using the last absolute shrinkage and selection operator (LASSO), and Receiver Operating Characteristic (ROC) curves were used to evaluate the signature. In addition, we analyzed downstream functional enrichment and immune infiltration to explore cuproptosis-inducing drugs and immunotherapeutic responses. All these analyses were validated using multiple datasets of the International Cancer Genome Consortium (ICGC).

**Results:**

TCA and copper malnutrition-related genes (*CDKN2A, IDH1, OGDHL, IDH3G, IDH3B, GLS, DLAT, LIPT1)* were finally included. According to the risk score, they were divided into high-risk and low-risk groups. Survival analysis showed that the overall survival (OS) of the high-risk group was significantly lower than that of the low-risk group. We established a risk prognostic feature to predict the OS of patients with HCC. Based on this feature and the clinical stage, we constructed a nomogram. Functional enrichment analysis revealed pathways related to organelle division and the cell cycle. Different risk scores had different immune abundances in immune cells (including macrophages and regulatory T-cells) and immune pathways (including antigen-presenting cells co-stimulation). Moreover, the drug sensitivity of eleschomol and PD-L1 in the high-risk group was better than that in the low-risk group. The status of *TP53* somatic mutation was also closely related to the risk score.

**Conclusion:**

In this study, we established a new prediction signature of eight genes related to cuproptosis and the TCA process, which can effectively predict the prognosis of HCC patients.

## Introduction

Liver cancer is the sixth most common type of malignant tumor worldwide (1), and hepatocellular carcinoma (HCC) is the most common subtype in the clinic, accounting for approximately 90% of cases. Liver cancer is known to have a very poor prognosis, and its high recurrence and mortality rates have been criticized. Therefore, it is urgent to develop a better prognosis prediction signature to evaluate the prognosis and provide a more accurate treatment plan.

Resistance to cell death is an important feature of tumors (2), and anti-tumor immunotherapy *via* induction of regulated cell death (RCD) is a new target for cancer therapy (3). Non-apoptotic RCD can be subdivided into autophagy, ferroptosis iron death cell pyrosis, and necroptosis (4, 5). Cuproptosis, a newly proposed RCD pattern, mediates cell death mainly through cytotoxicity caused by the mitochondria-dependent increase in energy metabolism and accumulation of reactive oxygen species (ROS) (6), and this pattern has been shown to be closely related to the tricarboxylic acid cycle (TCA). Tsvetkov’s study (7) showed that copper ions can directly bind to fatty acylated components in the TCA, which blocks the TCA, triggering protein toxic stress and inducing cell death. The decrease of TCA activity leads to the accumulation of acetyl CoA, which reduces DLAT activity and inhibits cuproptosis through competitive feedback inhibition. Both of which affect each other. Cancer cells can adjust their copper homeostasis mechanism by redistributing copper ions in the liver. The imbalance of copper homeostasis in HCC cells will lead to tumor progression (8) and drug resistance (9).Changes in gene expression of key enzymes in the TCA are closely related to the progression of HCC (10). Meanwhile, the Warburg effect in patients with liver cancer downregulates the TCA, resulting in the formation of a hypoxic microenvironment. The combined effect of cytotoxic TCA metabolites and gene mutations reduces cell differentiation, leading to the progression and metastasis of HCC (11). Hsu’s study also found that high TCA activity and mitochondrial respiration rate can increase the sensitivity of anti HCC drugs (12). Although some studies have revealed the impact of cuproptosis on HCC (13, 14), the close relationship between TCA and copper death shows that comprehensive research is more advantageous. Therefore, analyzing drug sensitivity of cuproptosis and the TCA gene, and effectively inducing cuproptosis to eliminate cancer cells will help provide new strategies for the treatment of HCC.

In this study, we identified copper poisoning and TCA-related genes that affect the prognosis of HCC, explored the characteristic function of each gene and its relationship with HCC, established a prediction model that affects overall survival (OS), and analyzed the guiding role of the prediction model for immunotherapy. In addition, we further explored the correlation between the prognostic model and other factors such as pathway analysis, immune score, and immune cell infiltration level. These works can provide more value and feasibility for the clinical treatment of HCC.

## Materials and methods

### Data source and processing

HCC mRNA expression and clinical data were obtained from The Cancer Genome Atlas (TCGA) database (https://tcga-data.nci.nih.gov/tcga/), including 345 patients. In addition, gene expression and clinical data of liver cancer patients were also downloaded from the International Cancer Genome Consortium (ICGC) database (https://dcc.icgc.org/projects/LIRI-JP), including 177 patients. Inclusion criteria included patients with gene expression and OS data. Gene expression data values were converted to log2 (TPM (transcripts per million) + 1) format. Baseline information of patients with HCC in the TCGA and ICGC datasets is shown in **Table 1**.

**Table 1:** Clinical characteristics of patients with liver cancer in two datasets.

Characteristics	TCGA cohort	LIRI-JP cohort
No. of patients	345	177
**Age (years)**		
Range	16-90	26-92
Median	61	66
**Gender**		
Female	108	81
Male	236	96
Unknown	1	
**AFP(ng/ml)**		
≤200	188	81
>200	73	96
Unknown	84	
**Vascular Invasion**		
Yes	102	81
No	189	96
Unknown	53	
**Stage**		
I	163	24
II	78	57
III	79	57
IV	3	39
Unknown	21	0
**Grade**		
I	53	
II	162	
III	112	
IV	12	
Unknown	5	177
**Overall survival outcome**		
live	221	104
death	124	73
**Overall survival (months)**		
Median	20.81	42.27

### Gene selection

Ten cuproptosis-related genes (7) including *CDKN2A, fdx1, DLD, DLAT, LIAS, GLS, LIPT1, MTF1, PDHA1, and PDHB* were first retrieved from previous studies. Moreover, 28 TCA-related genes were downloaded from the molecular signatures database, and 32 cuproptosis and TCA-related genes were included by taking the intersection. Then, Cox analysis and Kaplan-Meier (K-M) survival analysis were used to obtain 23 cuproptosis and TCA-related genes affecting OS for further study. The Log-rank method was used to test for statistical significance. The R packages used in the above analysis were the “survival” package and the “Survivor” package.

### Prediction signature and nomogram

To construct and evaluate signatures, TCGA cohort was used as the training set, and the ICGC data was used as the validation set. Based on λ, the “glmnet” software package was used for Lasso analysis of the 23 cuproptosis and TCA-related genes and OS data, and the following formula was used for calculation to establish a prognostic risk signature:


$$\sum_{i=1}^{\text{n}}\text{Coef }\left(Gene\right)\times \text{Expr (Gene)}$$

where Coef (Gene) is the coefficient of genes related to OS, and Expr (Gene) is the median of the corresponding gene expression characteristics according to the risk score. Using the median risk score as the cut-off value, patients were divided into a high-risk group (n = 158) and low-risk group (n = 157) in the TCGA main cohort. K-M and receiver operating characteristic (ROC) curves were used to test the accuracy of the prediction signature in predicting the prognosis of HCC patients. The ROC curve was drawn using the “timeroc” package, which also performs multivariate Cox analysis on TCGA and ICGC patients to determine whether the signature can predict the prognosis of patients independently of other factors. Finally, the RMS package was used to construct the nomogram and calibration chart.

### Cuproptosis-TCA signature transcriptome and genomic variation in hepatocellular carcinoma

The results of the differential gene expression analysis of eight prognostic features between TCGA liver cancer tissues and adjacent tissues, and the single-nucleotide variant (SNV) and copy number variation(CNV) analysis results of liver cancer samples were obtained from the GSCA website (http://bioinfo.life.hust.edu.cn/web/GSCALite/). The results of the correlation analysis of gene expression and IC50 values of antitumor drugs were obtained from the genomics of the cancer therapeutic response portal (CTRP) database.

### Functional enrichment analysis

LIMMA package was used to obtain the differentially expressed genes between the high-risk group and the low-risk group. Log | FC | > 1, adj. P< 0.05 was used as the screening criteria for significantly different genes. The differentially expressed genes were subjected to GO enrichment analysis and KEGG enrichment analysis using the R software package “clusterprofiler.” The results showed that Q< 0.05 was used as the screening standard.

### Single-nucleotide polymorphism (SNP) in the high and low-risk groups

The SNP data of HCC patients were obtained from the TCGA cohort. The maftools R software package was used to visualize the somatic mutation data of the high and low-risk groups according to the descending order of mutation, and the expression form is presented in a waterfall plot

### Immune infiltration analysis

The relative enrichment levels of 16 immune cells and 13 immune functions in HCC samples were calculated using the single-sample gene set enrichment analysis (SS GSEA) algorithm in R software with the “GSVA” package. The immunogenicity was then assessed using immunophenoscore (an indicator to measure the overall immunogenicity of tumors), which comprised of four modules, including effector cell (EC), immunosuppressive cell (SC), immune checkpoint (CP), major histocompatibility complex molecular(MHC). The higher the EC score, the greater the number of effector cells, and the higher the SC score, the lesser the number of immunosuppressive cells. The “IOBR” R package was used for the immunophenoscore analysis.

### Drug sensitivity analysis

According to the drug sensitivity results in the Cancer Genome Project (CGP) database of the cancer genome project, the estimated IC50 of TCGA and ICGC array samples for multiple drugs in CGP was calculated using the “prophetic” package. The test was performed according to the high-risk and low-risk groups to observe the difference between both groups based on drug sensitivity. In addition, the correlation coefficient between IC50 and the risk score of each sample was calculated.

### Correlation analysis of immunotherapy

The response to immunotherapy of different risk groups in the imvigor210 (MUC) cohort was discussed. Advanced urothelial cancer (imvigor210 cohort) anti-PDL1 immunotherapy array was downloaded from http://research-pub.gene.com/IMvigor210CoreBiologies. We calculated the risk score of each sample of the imvigor210 array and compared the difference in risk scores between the immune treatment response group and the non-response group. The “ROC” package was used to draw the ROC curve of the risk score predicting the immune treatment response. The samples were divided into high-risk and low-risk groups according to the risk score using the best cut-off for K-M survival analysis.

### Statistical analysis

All analysis in this study were performed using R language (4.0.5). Rank-sum test or Kruskal–Wallis test was used to compare quantitative data between two or more groups. Spearman correlation coefficient was used for correlation analysis. For K-M survival analysis, the log-rank method was used for the statistical test. P< 0.05 was considered statistically significant. The Benjamin Hochberg method was used to calibrate the p value and reduce false positives.

## Results

### Construction of cuproptosis and TCA-related prognosis signature

**Figure 1:**
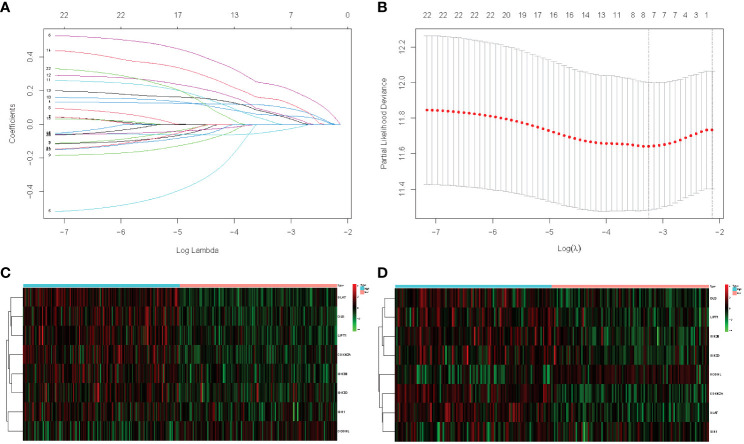
**(A)** LASSO coefficient profiles of the 23 cuproptosis and TCA-related genes from the TCGA cohort. **(B)** Partial-likelihood deviance of variables revealed by the LASSO regression signature. Red dots represent the partial likelihood of deviance values, gray lines represent the standard error (SE), and the two vertical dotted lines on the left and right represent optimal values by minimum criteria and 1–SE criteria, respectively. **(C)** TCGA heatmap of the prognostic signature consisting of eight cuproptosis and TCA-related genes. **(D)** ICGC heatmap of the prognostic signature consisting of eight cuproptosis and TCA-related genes.

In this study, 345 HCC cases from TCGA were selected as the training cohort, and 177 HCC patients from the ICGC dataset were used as the validation cohort. The detailed clinical characteristics of the two cohorts are summarized in **Table 1**. We used LASSO Cox regression analysis to demonstrate that eight genes had the highest predictive value of OS for HCC, and screened out eight genes that affect the OS prognosis of HCC patients based on the value of λ (**Figures 1A, B**), using the following formula to establish a prognostic signature related to cuproptosis: Risk score = (−0.04 × OGDHL+0.23 × DLAT+0.11 × CDKN2A+0.05 × GLS+0.06 × IDH3B+0.02 × IDH3G+0.01 × IDH1+0.15 × Lipt1). HCC samples were divided into high-risk group (n = 172) and low-risk group (n = 173). Then, we explored the differential expression of related genes in the signature in the high-risk and low-risk groups. OGDHL gene was highly expressed in the low-risk group, and other genes were highly expressed in the high-risk group (**Figures 1C, D**).

### Differential expression and variation of genes

**Figure 2:**
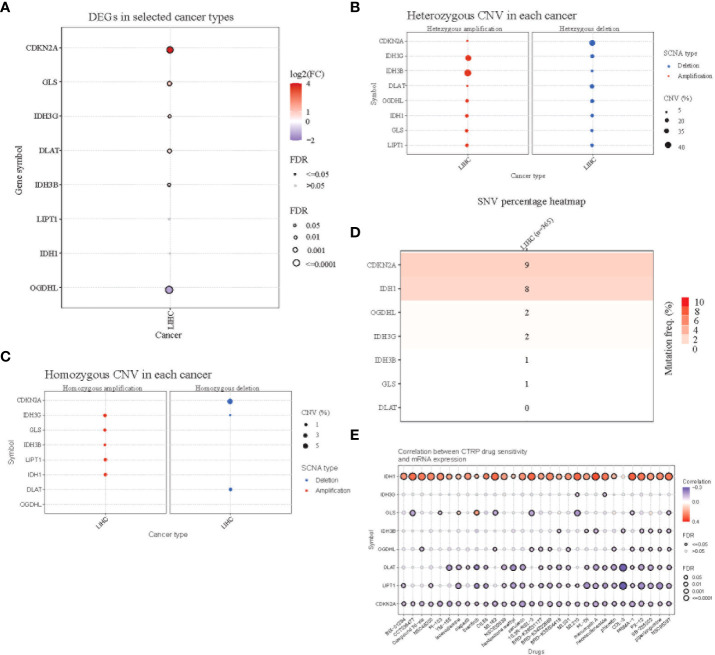
Mutation analysis of the 8-gene signature. **(A)** Differential expression of the eight genes in TCGA HCC tissues and normal adjacent tissues. Red(log2FC>1) indicates high expression, while blue indicates low expression. **(B)** Homozygous copy number variation of the eight genes in the TCGA HCC cohort. Amplifications are shown on the left and deletions are shown on the right. **(C)** Heterozygous copy number variation of the eight genes in the TCGA HCC cohort. **(D)** Single nucleotide variant (SNV) variation of the eight genes in the TCGA HCC cohort. **(E)** Correlation analysis of the expression of the eight genes and drug sensitivity using the CTRP database.

Comparing the differential expression of eight genes in HCC and normal tissues, *CDKN2A* showed significantly higher expression, while the expression of *OGDHL* in HCC tissues was lower than that in normal tissues (**Figure 2A**). Subsequently, we studied the CNVs and SNVs of eight genes in the TCGA liver cancer cohort. The gene with significant deletion of CNV was *CDKN2A*, and the gene with the highest CNV amplification was *IDH3G* (**Figures 2B, C**). Regarding SNV variation, the mutation frequency of *CDKN2A* and *IDH1* was significantly higher than that of other genes (**Figure 2D**). Finally, drug sensitivity analysis was carried out using CTRP database. It was found that the high expression of *IDH1* gene was the main gene causing drug resistance. The high expression of *DLAT, LIPT1, and CDKN2A* genes was negatively correlated with IC50 values (**Figure 2E**).

### Survival analysis

**Figure 3:**
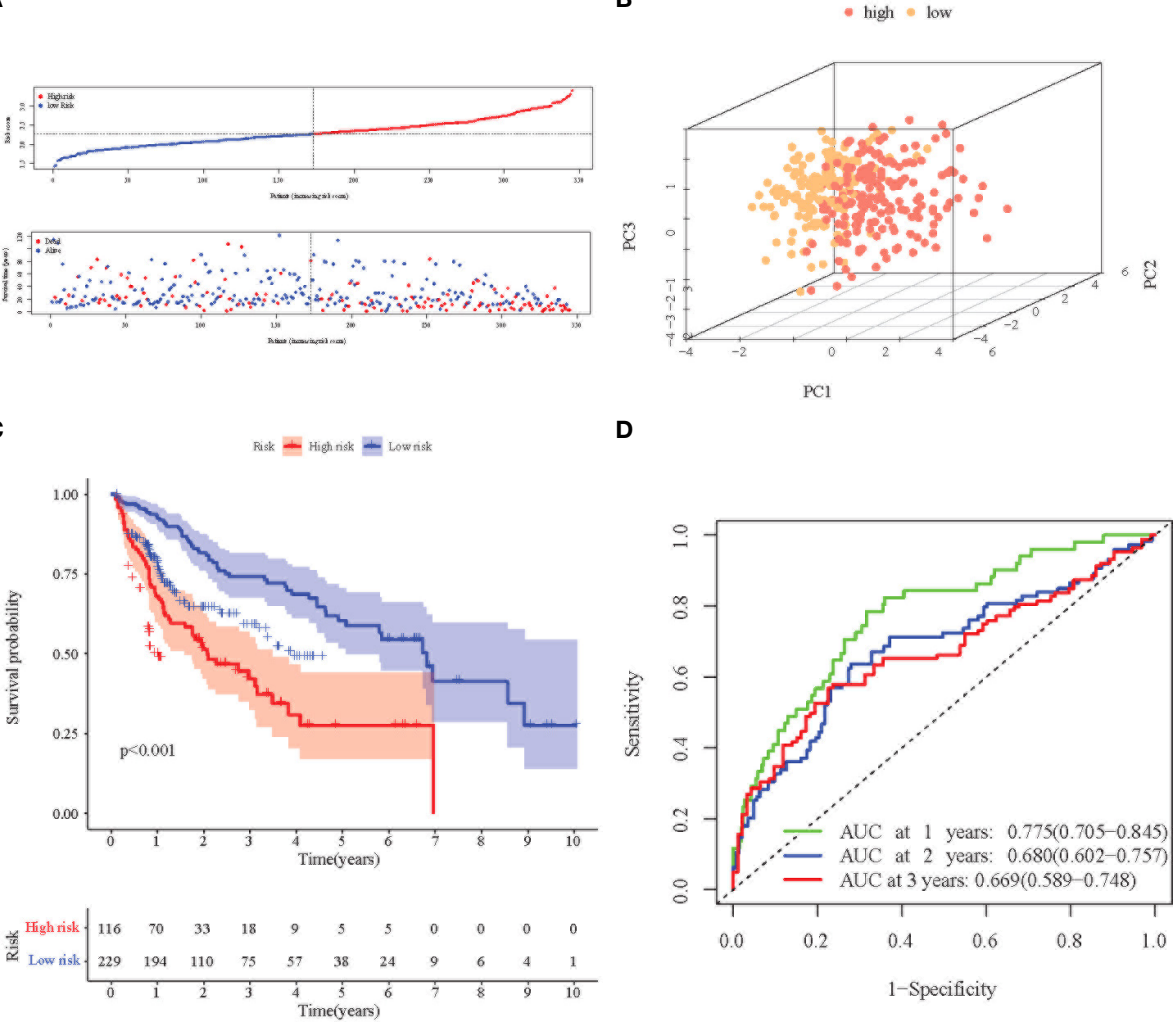
Prognostic analysis of the 8-gene signature in the TCGA cohort. **(A)** Distribution of risk score in high-risk and low-risk groups of the TCGA cohort; and distribution of overall survival in the TCGA cohort in different groups. **(B)** 3D-PCA analysis of the TCGA cohort. **(C)** Kaplan–Meier curve of the 8-gene signature in the TCGA cohort (P<0.05). **(D)** ROC analysis for risk signature at 1, 2, and 3 year survival time in the TCGA cohort.

**Figure 4:**
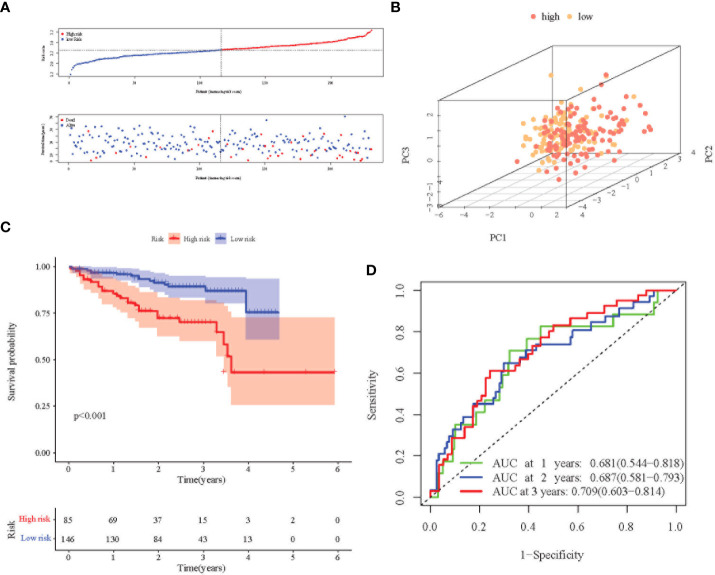
Prognostic analysis of the 8-gene signature in the ICGC cohort. **(A)** Distribution of risk score in high-risk and low-risk groups of the ICGC cohort; and distribution of overall survival in the ICGC cohort in different groups. **(B)** 3D-PCA analysis of the ICGC cohort. **(C)** Kaplan–Meier curve of the 8-gene signature in the ICGC cohort (P<0.05). **(D)** ROC analysis for risk signature at 1,2, and 3 year survival time in the ICGC cohort.

Principal Component Analysis (PCA) analysis was performed according to the gene expression level to compare the differences between the high-risk group and the low-risk group. The prognosis signature can clearly divide HCC patients into a high-risk group and a low-risk group (**Figures 3A, B**, **4A, B**). The distribution of the two groups on the PCA plot are relatively scattered. Kaplan–Meier survival analysis was subsequently performed on the 345 patients in the TCGA cohort, and patients with higher risk scores were associated with higher risk of death and shorter survival time (**Figure 3C**, p< 0.001). Similar results were also obtained in the high-risk group (n = 115) and low-risk group (n = 116) among the 231 patients in the ICGC cohort (**Figure 4C**, p< 0.001). ROC analysis evaluated the predictive effect of risk score on OS. The area under the curve (AUC) of 1, 2, and 3 years in the TCGA was 0.775, 0.680, and 0.669 respectively (**Figure 3D**), and the AUC of 1, 2, and 3 years in the ICGC cohort was 0.681, 0.687 and 0.709, respectively (**Figure 4D**), proving that the prognostic features have good accuracy, specificity, and clinical applicability, and can accurately predict the prognosis of HCC patients.

**Figure 5:**
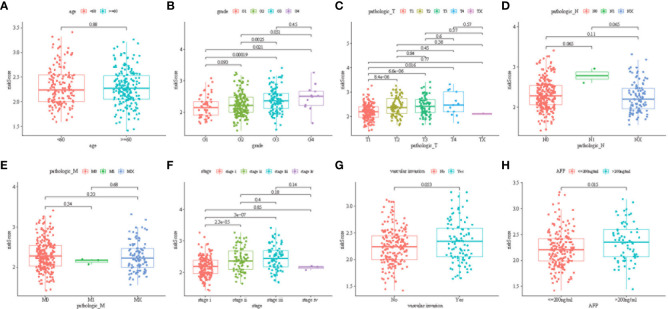
Distribution of risk scores among age group **(A)**; tumor grade **(B)**; tumor pathologies T **(C)**, N **(D)**, and M **(E)**; cancer stage group **(F)**; vascular invasion **(G)**; and AFP group **(H)** (P < 0.05).

**Figure 5** illustrates the correlation between the prediction signature of TCGA cohort and the clinical characteristics of HCC. We found that higher risk score was positively correlated with various adverse clinicopathological characteristics. The risk score of T2 and above stages was significantly higher than that of the T1 stage (**Figure 5C**, p< 0.05). The risk score also increased with stages I, II, and III (**Figure 5F**, p< 0.05), and was significantly correlated with the size of alpha-fetoprotein(AFP) (**Figure 5H**, p< 0.05). There was no statistical difference in other clinical features including age (**Figure 5A**), vascular invasion (**Figure 5G**), N stage (**Figure 5E**), and M stage (**Figure 5F**) (p > 0.05).

**Figure 6:**
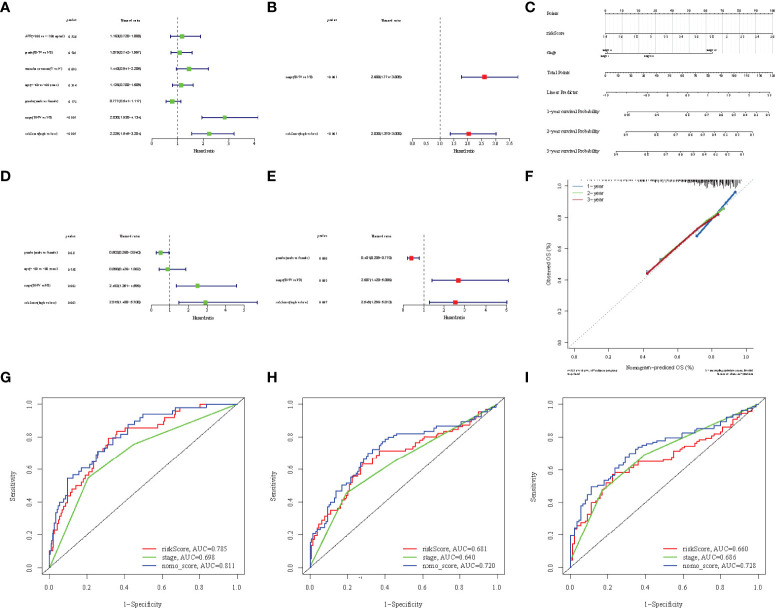
Univariate Cox regression analyses of overall survival in the TCGA **(A)** and ICGC **(D)** cohorts, and multivariate Cox regression analyses of overall survival in the TCGA **(B)** and ICGC **(E)** cohorts. **(C)** Nomograms for predicting 3-year survival in the TCGA cohort. **(F)** Calibration curves for the nomograms the TCGA cohort. **(G-I)** ROC curves for 1, 2, and 3-year time showing the comparison between the survival prediction ability of each nomogram and other risk factors.

To promote the clinical application of the prediction signature, we integrated the clinical information of TCGA patients. Univariate and multivariate Cox regression analysis showed that risk score (p< 0.001, HR = 3.711, 95% CI: 2.557–5.384) and liver cancer stage (p< 1.001, HR = 3.639, 95% CI: 2.510–5.277) were independent prognostic factors for OS (**Figures 6A, B, D, E**).The relevant features were integrated to construct nomograms to predict the one-year, two-year, and three-year OS of HCC patients (**Figure 6C**). The calibration curves of the one-year, two-year, and three-year OS nomogram showed good consistency between the predictions and the actual observations (**Figure 6F**). Compared with other prognostic factors, we also found that the AUC of the one-year, two-year, and three-year time ROC analysis was 0.811, 0.720, and 0.728 (**Figures 6G–I**), respectively, which was higher than the AUC of risk score (0.785, 0.681, and 0.66) and stage (0.698, 0.64, and 0.686). The predictive performance of the nomogram was significantly higher than that of other prognostic features. The above results indicate that the risk signature can be used as an independent prognostic factor and can also be combined with existing clinical indicators.

### Functional enrichment analysis

**Figure 7:**
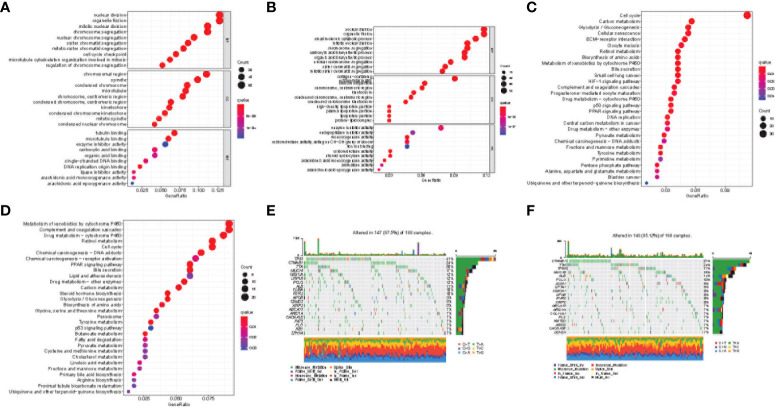
Functional enrichment of the cuproptosis and TCA-related genes between risk groups. Results of GO enrichment analyses in the TCGA **(A)** and ICGC **(B)** cohorts. Results of KEGG enrichment analyses in the TCGA **(C)** and ICGC **(D)** cohorts. The 20 genes with the highest mutation frequency in the high-risk **(E)** and low-risk **(F)** groups of the TCGA cohort.

Based on the risk signature, we studied the differences in gene functions and related pathways among different risk groups and explored the biological processes related to the cuproptosis and TCA-related genes. We analyzed the relevant pathways through the GO and KEGG databases. GO enrichment analysis revealed the main pathway of nuclear division and organelle fission (**Figures 7A, B**). Moreover, in the KEGG pathway enrichment analysis, the main related pathways included cell cycle, P450, and complement and coordination Cascades (**Figures 7C, D**). To clarify whether there were differences in gene mutations between the high-risk group and the low-risk group, we downloaded and analyzed the SNP variant data of TCGA, and missense mutations were the most common mutation type in HCC patients. T > A ranked the highest in the single nucleotide variant (SNV) category. TP53 (41%) and *CTNNB1* (24%) showed a higher mutation frequency than other genes in the high-risk group, and *CTNNB1* had a higher mutation frequency in the low-risk population (**Figures 7E, F**).

### Immune feature analysis of cuproptosis and TCA-related gene signature

**Figure 8:**
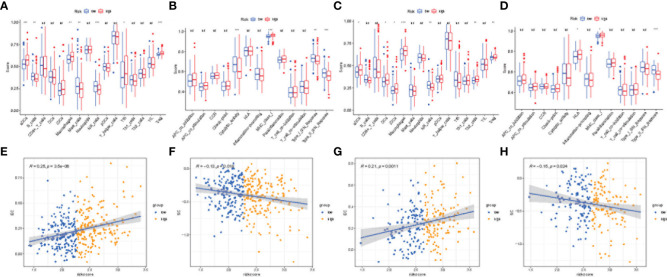
Immune microenvironment and immunophenoscore analyses. Box plots showing immune cell infiltration fraction and immune function activity in TCGA **(A, B)** and ICGC **(C, D)** cohorts. Correlation plot for effector cell (EC) score and risk score in TGCA **(E)** and ICGC **(F)** cohorts. Correlation plot for immunosuppressive cell (SC) score and risk score in TCGA **(G)** and ICGC **(H)** cohorts. (***p < 0.001, **p < 0.05, *p < 0.1, ns, no significance).

The differences in immune status between the high-risk and low-risk populations were further discussed, and we estimated the degree of immune cell enrichment and the scoring path of immune-related functions (**Figures 8A–D**). In the TCGA cohort, follicular helper T cells, macrophages, and Tregs were significantly upregulated. Regarding the immune pathway, the high-risk group was significantly enriched in antigen-presenting cell (APC) co-stimulation, APC co-inhibition, parainflammation, and the endogenous pathway (MHC-1). The activation of immune components in the tumor microenvironment and oncogenic pathways activated by immune pathways may lead to worse prognosis of patients in the high-risk group. Then, we described the immunogenicity of the tumor by immunophenotypic scoring. Effector cell (EC) was highly expressed in the high-risk group (**Figures 8E, G**; p< 0.05), but the immunsuppressive cell (SC) score was lower (**Figures 8F, H**; p< 0.05). The above results indicate that the high-risk group has more immune effector cells and fewer immunosuppressive cells and has a stronger ability to regulate the immune edge and immune microenvironment. However, there was no statistical difference in checkpoint score and antigen presentation score (p > 0.05, **Supplementary Figure S1**).

### Drug sensitivity analysis

**Figure 9:**
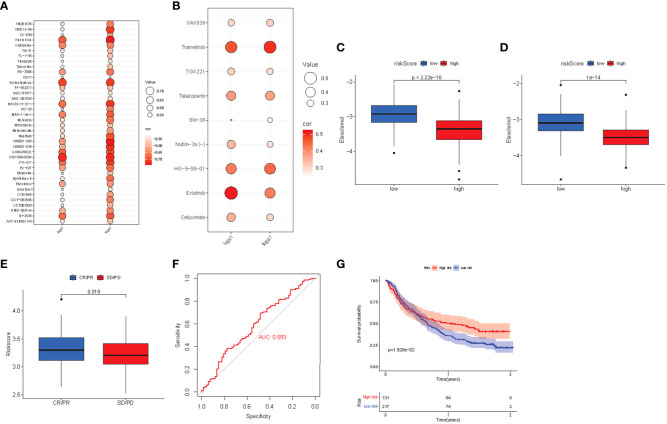
**(A)** Bubble chart of negative correlation between estimated IC50 value and risk score of 38 drugs. **(B)** Bubble chart of positive correlation between estimated IC50 value and risk score of 9 drugs. **(C)** Box plot of the estimated IC50 values of eleshomol in the high-risk and low-risk groups of the TCGA cohort. **(D)** Box plot of the estimated IC50 values of eleshomol in the high-risk and low-risk groups of the ICGC cohort. **(E)** Box plot of correlation between immunotherapy and risk score in imvigor210 cohort. **(F)** ROC curve of risk score to predict the effect of immunotherapy. **(G)** K-M survival curve of immunotherapy prognosis in different risk groups.

Poor prognosis is often closely related to drug resistance. We predicted the resistance response of the two risk subgroups to common drugs and calculated the IC50 of multiple drugs in the TCGA and ICGC array samples cancer genome project (CGP). The screening criteria were p< 0.01 and correlation coefficient< -0.5. We found that in TCGA and ICGC arrays, the estimated IC50 and risk score of 38 drugs were significantly negatively correlated (**Figure 9A**), and the IC50 of the high-risk group was lower. Moreover, the IC50 and risk score of 9 drugs were also significantly positively correlated (p< 0.01), while the low-risk group had a lower IC50 (**Figure 9B**). Among the drugs was eleschomol, a cuproptosis inducing drug. We compared the IC50 values of eleschomol in different risk groups, and the high-risk group had a better sensitivity (**Figures 9C, D**; p< 0.05).

Regarding the drug analysis of targeted therapy, the high-risk group was more sensitive to targeted drugs (including tivozanib, mastinib, and crizotinib) than the low-risk group. Subsequently, we analyzed sorafenib, linifanib, and other drugs, and found that the high-risk group still had significant sensitivity, while the low-risk group had better drug sensitivity for trametinib and erlotinib, which can be further studied in the future.

Finally, the risk score of patients was calculated in the anti-PDL1 immunotherapy array of advanced urothelial carcinoma (imvigor210 cohort), and the prognosis level of patients was evaluated according to the m-resist criteria. Patients with partial and complete remission after immunotherapy had higher risk scores (**Figure 9E**). The ROC curve verified that the risk score had a good predictive effect on the response to immunotherapy (AUC = 0.593, **Figure 9F**). Imvigor210 array patients were divided into high-risk group and low-risk group according to the critical risk score. The K-M curve found that the prognosis of the high-risk group was better (**Figure 9G**). Thus, the high-risk group is more suitable for anti-PDL1 immunotherapy.

## Discussion

The liver is an important organ for storing and metabolizing copper, and the imbalance between copper deposition and copper excretion often leads to liver injury, even liver cancer (9). Overloaded copper ions in HCC can induce cell death by targeting lipoacylated TCA circulating proteins (7). TCA can promote HCC growth through metabolite biological abnormalities (15, 16). However, few studies have discussed the predictive value of cuproptosis and TCA-related genes for HCC prognosis. In this study, eight genes related to copper dystrophy and the TCA process were included. These genes are significantly related to OS of HCC patients and have a very important predictive value. Therefore, we established a clinical prognosis signature based on the TCGC cohort and verified the predictive efficiency with the ICGC cohort.

The risk signature finally includes TCA and cuproptosis related genes (DLAT, GLS, LIPT1, CDKN2A, IDH3B, IDH3G, IDH1, OGDHL). DLAT, CDKN2A and GLS are genes regulating PDH expression and LIPT1 is genes regulating protein fatty acylation during cuproptosis. IDH and OGDHL genes are important genes regulating TCA process. The down-regulation of OGDHL and IDH genes in liver cancer tissue will lead to the metabolic activity of the tricarboxylic acid cycle, even reversal of the TCA cycle (17). Tumor cells can rely on reverse TCA cycle to produce large amounts of citric acid and acetyl coenzyme a, which can promote tumor proliferation and affect the process of cuproptosis. CDKN2A is a negative regulator of cuproptosis. Copy number deletion and SNV high mutation are the causes of differential expression between HCC and normal tissues. *CDKN2A* regulates the cell cycle and stops at the G phase, leading to unlimited cell proliferation (18–20). Compared with normal tissues, the expression of *OGDHL* is significantly reduced in HCC, which promotes the metabolism of glutamine (21, 22). Glutamine acts as a mitochondrial energy source to provide energy to cancer cells. At the same time, the upregulation of *glutamylase* (GLS) gene expression also converts glutamine into glutamate, thus promoting the growth and metastasis of cancer cells. *IDH* gene mutations lead to metabolic rearrangements that affect cellular redox homeostasis. *IDH* mutations are mainly copy number amplification and SNV high mutations, and the mutated *IDH* catalyzes α-KG and generates 2-hydroxyglutaric acid (2-HG) (23), which leads to epigenetic and energy metabolism abnormalities (24) and promotes the proliferation of liver cancer. Meanwhile, some studies have found that IDH3a promotes epithelial mesenchymal transition (EMT) in HCC cells by regulating the expression of MTA1 (25). The catalytic action of DLAT is the only way for pyruvate to be converted to acetyl CoA after entering the mitochondria (26). Its upregulation promotes ATP production and catabolic responses and promotes tumor cell growth and proliferation (27). A new prognostic signature was then established. We first verified the predictive effect of the prognostic risk score signature based on the cuproptosis gene, and then combined the signature with the liver cancer stage to further develop a nomogram for predicting the prognosis of HCC patients. Compared with risk score and other clinical features, the nomogram can better predict the one-year, two-year, and 3-year survival rate of HCC. External validation also confirmed the potential of the nomogram. We compared the previously established cuproptosis signatures. Including 6 lncRNA and 3 mRNA models established by Liu et al (13); 6 lncRNA signatures related to cuproptosis established by Zhang et al (14);and 16 prognosis signatures related to cuproptosis genes established by Fu et al (28). Although all the above signatures can accurately predict the prognosis of patients, the combination of TCA and cuproptosis related signatures is more comprehensive in terms of metabolism and genetics, and anti-tumor drugs are more sensitive to cells with higher mitochondrial metabolism and TCA activity.

Functional analysis showed that these genes rely on biological pathways and functions related to the cell cycle, organelle fission, P450, and the complement pathway, and p53 and CDKN2A lead to disorder of cell cycle regulation and may lead to uncontrolled cell proliferation (29, 30). Mitochondria are the workplace of TCA, and their dynamics are unbalanced. The enhancement of the mitochondrial cleavage state can inhibit mitochondria-dependent apoptosis (31), enhancing the Warburg effect (32), and promoting the proliferation of liver cancer cells (33). Anaphylatoxins C3a and C5a in the complement cascade can increase mitotic signaling pathways, induce angiogenesis together with M2 macrophages, and create an immunosuppressive microenvironment (34–37). Inhibiting or blocking these pathways could be a potential treatment target for liver cancer in the future. The gene with the highest mutation frequency in the high-risk group is *TP53*, which is the most significant difference from the low-risk group and the middle risk group. It has been found that p53 interacts with *puma* gene in HCC, inhibits mitochondrial uptake of pyruvate, forces organelles to stop respiratory function, and reduces copper-induced cell death (7), which promote the enhancement of cancer cell metabolism and the function of copper-induced drugs. The mutation frequency of *CTNNB1* in the low-risk group is the highest in the population, and the mutation of *CTNNB1* may be the main cause of immunotherapy rejection (38, 39).

The roles of cuproptosis in the tumor immune microenvironment are still unclear. We explored the relationship between genes and tumor-infiltrating immune cells in the signature. The high-risk group had a high proportion of macrophages, Treg cells, and dendritic cells. Macrophages secrete CCL22 to induce Treg migration to the tumor area and inhibit the activation of CD4 (+) CD25 (-) T cells, resulting in poor prognosis in the high-risk group (40). Direct immunosuppression of macrophages can mediate immune escape through the PD-1/PD-L1 axis and promote the expression of the immunosuppressive molecule PD-L1 (41–43). Meanwhile, PD-1/PD-L1 inhibitors can promote the pro-inflammatory polarization of macrophages and limit the spread and metastasis of tumors (44, 45). In the study of immune pathways, the expression of MHC-1 differs according to risk groups and plays an important role in immune evasion and immune surveillance (46, 47). Another parainflammatory pathway, as a possible pathway, has been proven to be the main driving force of *TP53* mutation (48). The change in immune microenvironment is very important for PD-L1 inhibitor treatment to obtain the optimal antitumor immune response. Therefore, we selected the gene expression profile and clinical features from the immunotherapy cohort (imvigor210) of urothelial carcinoma (UC) treated with anti-PD-L1 drugs to study the relationship between the constructed risk signature and immune response. In this anti-PD-L1 cohort, patients with high-risk scores who received PD-L1 inhibitor therapy showed significant clinical benefits and significantly prolonged survival. Therefore, PD-L1 inhibitors are more suitable for the high-risk group.

Cuproptosis is a new type of cell death, and future drug-induced therapy may provide a new scheme for liver cancer. Eleschomol, a drug for cuproptosis, causes cuproptosis in cells by transporting copper ions to the mitochondria (49). In this study, we evaluated the drug sensitivity of eleschomol and found that it has a better prognosis in high-risk groups. Regarding the most common targeted therapy in the treatment of liver cancer, the benefit of targeted therapy in the high-risk group is higher than that in the low-risk group, which can be further studied in the future.

Our study has several limitations. The clinical features extracted from the TCGA and ICGC databases are limited and incomplete. This study is a retrospective study and requires an independent prospective cohort to validate the prognostic signature developed in the present study. The value of these genes as potential pharmacological targets also needs further study. Further clinical experiments are also needed; therefore, further *in vivo* or *in vitro* experiments will be carried out in the later stage.

## Conclusion

A prognostic signature composed of eight cuproptosis and TCA-related genes was constructed by various bioinformatics methods, and a nomogram was developed in combination with liver cancer staging, which showed a good predictive value for the prognosis of liver cancer. The high-risk group received copper-induced drugs and PD-L1 inhibitors, which may provide some basis for the individualized treatment and evaluation of liver cancer patients.

## Data availability statement

The datasets presented in this study can be found in online repositories. The names of the repository/repositories and accession number(s) can be found in the article/**Supplementary Material**.

## Author contributions

Conceptualization, QL and HZ. Methodology, QZ and YZ. Software, QZ. Validation, HZ. Formal analysis, QZ and LM. Writing - Original Draft, QZ and SL. Writing – Review and Editing, QL. Visualization, YZ. All authors have read and agreed to the published version of the manuscript. All authors contributed to manuscript revision, read, and approved the submitted version.

## Conflict of interest

The authors declare that the research was conducted in the absence of any commercial or financial relationships that could be construed as a potential conflict of interest.

## Publisher’s note

All claims expressed in this article are solely those of the authors and do not necessarily represent those of their affiliated organizations, or those of the publisher, the editors and the reviewers. Any product that may be evaluated in this article, or claim that may be made by its manufacturer, is not guaranteed or endorsed by the publisher.

## Supplementary material

Supplementary Figure 1
**(A)** Comparison of major histocompatibility complex (MHC) molecular score (P>0.05). **(B)** Comparison of immune checkpoint (CP) score (P>0.05).
