# Supplementary figures and images for: A prognostic signature of cuproptosis and TCA-related genes for hepatocellular carcinoma
Source: Front Oncol. 2022 Oct 17;12:1040736. doi: 10.3389/fonc.2022.1040736 (PMC9619237; doi:10.3389/fonc.2022.1040736)

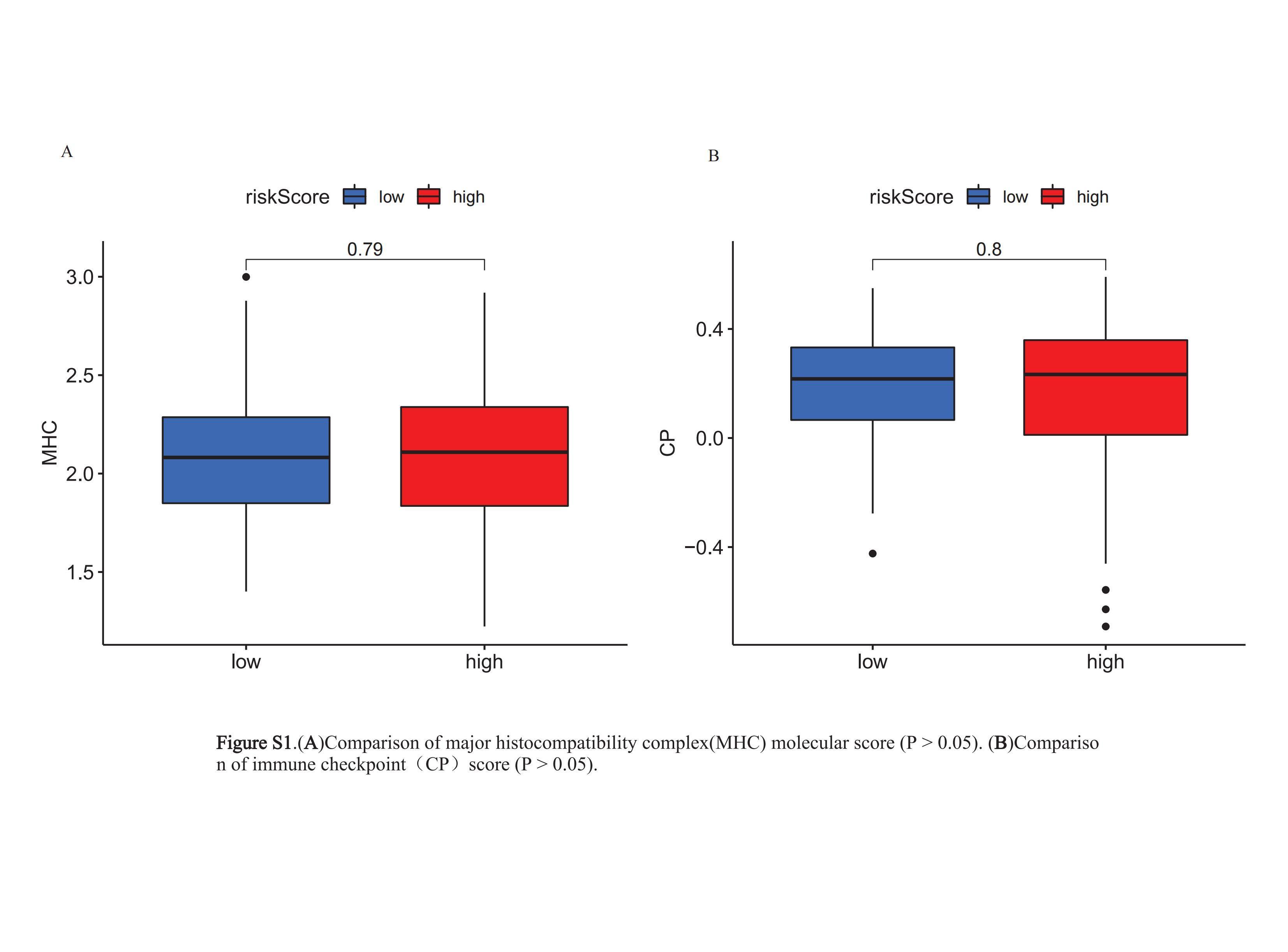

Supplement: Supplementary Figure 1 — (A) Comparison of major histocompatibility complex (MHC) molecular score (P>0.05). (B) Comparison of immune checkpoint (CP) score (P>0.05). [file Image_1.jpeg]
